# Sustained Reduction in Intravenous Pump Turnaround Time Using Lean Methodology

**DOI:** 10.1097/pq9.0000000000000585

**Published:** 2022-08-01

**Authors:** Smriti Neogi, Glenn Schneider, Joshua K. Schaffzin

**Affiliations:** From * James M. Anderson Center for Health Systems Excellence, Cincinnati Children’s Hospital Medical Center, Cincinnati, Ohio; † Department of Clinical Engineering, Cincinnati Children’s Hospital Medical Center, Cincinnati, Ohio; ‡ Division of Infectious Diseases, Cincinnati Children’s Hospital Medical Center, Cincinnati, Ohio; § Department of Pediatrics, University of Cincinnati College of Medicine, Cincinnati, Ohio.

## Abstract

Intravenous pumps provide essential, life-sustaining medications to patients. Pumps must be in working order and available on short notice to be effective. We identified inefficiencies in our pump management process that inflated the cost and time to complete repairs.

**Methods:**

Our multidisciplinary team completed a 60-day before-after trial that followed the Toyota Production System Lean methodology and evaluated the sustainability of our improvements for the following 48 months. We used value stream mapping and manual time studies to identify areas for improvement. Device turnaround time (TAT) was the number of days from receiving a device for repair to its return to service. Interventions included: establishing a reliable system to receive and track repair requests, creating a better organized, more efficient workroom, streamlining the inventory of repair parts, and tracking delivery systems reliably.

**Results:**

We reduced mean intravenous pump TAT by 89% and sustained TAT at 74%–97% below baseline for 4 years, including during the COVID pandemic.

**Conclusions:**

We used Lean methodology to create a system to receive, track, and provide safe, functional equipment to providers promptly. Both clinical and nonclinical healthcare professionals can use Lean to produce a sustainable improved system.

## INTRODUCTION

Intravenous (IV) pumps are frequently part of medical care provided in an acute setting. Over time, pumps have become more automated and sophisticated to deliver proper dosing and prevent errors. At the same time, they have become more technically intricate, liable to fail, and are subject to recalls. In 2020, 10 of 32 US Food and Drug Administration medical device recalls were related to infusion pumps.^1^ Staff who use, repair, and maintain the equipment are frequently educated and re-educated on processes, and unexpected events and recalls challenge staff and systems. Perceived frequent malfunction, poorly sustained function, and stories of critical failures can contribute to providers attempting to circumvent established processes or use older devices, which may increase the risk of error and patient harm.

In 2016, we identified a pattern of dissatisfaction with our IV pumps submitted for repair. Providers complained that they required corrective maintenance frequently, the time for a pump to undergo repair was excessive, and they could not rely on timely delivery of pumps when needed. Our Clinical Engineering Department (CE) responded by increasing personnel and availability of repair materials and purchasing additional devices. However, this did not resolve the issue and generated a higher cost. Additionally, we identified that units sometimes held on to pumps they thought worked rather than sending them to CE for routine maintenance and updates. Thus, we initiated an improvement effort using Lean methodology to decrease the median pump turnaround time (TAT) from 37.5 to 15 days within 2 months of the project’s start.

## METHODS

### Setting

Cincinnati Children’s Hospital Medical Center (CCHMC) is a large, free-standing academic medical center serving patients and families who live locally, nationally, and abroad. The CE Department is tasked with supporting the busy clinical services at two full-service hospitals and nine neighborhood locations, including five urgent care clinics. During the fiscal year 2019, there were 33,145 admissions, 173,495 emergency and urgent care visits, 35,987 surgeries, and 1,028,686 ambulatory visits. During the fiscal year 2020 and the COVID pandemic, volumes remained at 87%–90% 2019 levels, and ambulatory visits increased by 5%. CCHMC has a long-standing commitment to improving science education and implementation.^2^ Beginning in March 2016, CCHMC initiated a Lean Collaborative to teach and apply Lean principles across the organization. Since then, an average of four teams per 29 cycles have completed project-based 60-day training.^3^

CE supports the organization from a centralized model with technicians of varying levels of experience and expertise. Our project focused on corrective rather than preventive maintenance, for which CE is responsible for all devices in the hospital. On average, CE repairs about 3,500 pumps annually.

### Planning the Intervention

**Fig. 1.**
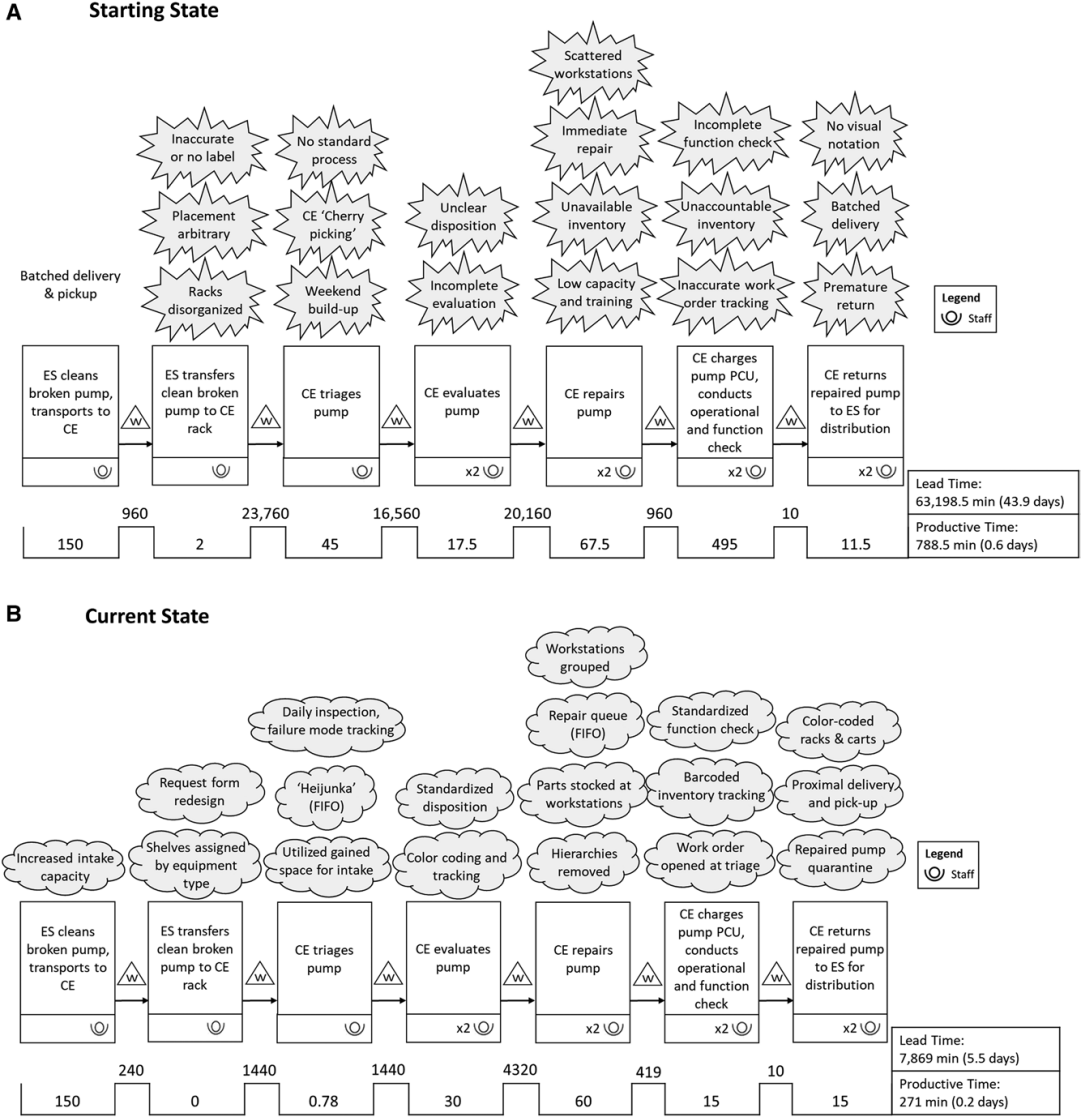
Value stream maps. A, Starting state value stream map. Potential nonvalue-added work was identified (see Table 1) and represented by an “angry cloud” over the appropriate process step. B, Current (final) state value stream map. Interventions to “angry clouds” are represented by “fluffy cloud” (see Table 1). Mean times in minutes are displayed below the process. ES, environmental services; FIFO, first-in-first-out; PCU, programmable control unit.

The Lean team members suspected that delays were occurring in both lead times, defined as the time between receiving a request and returning a pump into circulation, including idle time between process steps, and process time, defined as the amount of time the staff worked on the request. To understand the current process and lead times, the team conducted observations and time studies to create a value stream map (Fig. 1A) of the current state of the process to identify Kaizens—opportunities for small, continual improvements based on the perspective and knowledge of people doing the work.^4^ An improvement summary was created by identifying areas of opportunity (Table 1).

**Table 1.** Improvement Summary

Process Step		Improvement Opportunity (Angry Cloud)^*^	Improvement Intervention
Step 1	ES cleans broken pump, transports to CE on “Clean Cart”	ES batches pickup and delivery for select items	Improved intake capacity
Step 2	ES transfers clean broken pump to “HallwayTriageRack”	Pumps labeled incorrectly or not at all	Redesigned request forms to capture complete information and customer concerns
		Pumps placed on rack haphazardly	Designated shelves by equipment type
		Racks not organized	
Step 3	CE triages pump	No standardized process	Daily inspection of all IV pumps on rackStreamlined work order creationBarcode scanning for failure modes
		CE avoids “difficult” devices and chooses which pump to work on (“cherry picking”)	“Heijunka”—changed process to meet demand and not technician preference—FIFO
		Weekend buildup increases work for the following week	Space gained with workstation organization (step 5) utilized for intake
Step 4	CE evaluates pump	Pump disposition decision unclear	Standardized process
		Evaluation incomplete	Color coded cartsBegan tracking devices submitted for repair
Step 5	CE repairs pump	Workstations scattered throughout workspace	Grouped workstations together and organized to be identical
		Pump repairs begin immediately after triage and evaluation	Pumps placed in repair queue (FIFO)
		Parts unavailable, inventory levels not maintained	Frequently used parts stocked at workstations to expedite repairInventory tracking with barcodes
		Low capacity and training to repair pumps	Removed existing hierarchies by having leaders engage staff directly to solve problems
Step 6	CE charges PCU, conducts operational and function check	Operational and function check may be incomplete	Standardized process
		Inconsistent part accountability	Inventory tracking with barcodes
		Work orders created and completed at end of repair process (TAT not measured accurately)	Work orders opened during triage (step 3) to document accurate TAT
Step 7	CE returns repaired pump to ES for cleaning and distribution	No visual notation of complete vs incomplete repairs	Color-coded racks and carts
		Batched delivery of completed pumps	Pump delivered soon after repairCE pick up of clean broken pumps when delivering repaired pumps daily in addition to ES daily drop-off (step 2)
		Pumps returned prematurely (eg, safety report submitted after return)	Pump quarantine period

^*^Also referred to as Kaizen Burst.

ES, environmental services; FIFO, first-in-first-out.

### Interventions

Our primary customers were clinical providers using the IV pumps for patient care. Hospital operations and finance staff were also customers, given the costs generated by repair work, equipment purchases, and lost clinical revenue. We also considered patients and families customers because system function impacts care quality. We elicited informal feedback from all customers and reviewed formal complaints to identify common themes and improvement opportunities.

We modified our process in small increments (Kaizens) to enable efficiency and accurate tracking. We engaged all CE staff directly through bi-monthly departmental meetings and weekly huddles in the workspace at shift changes. At least one and often numerous members of the core project team and departmental leadership attended all meetings and huddles. We elicited feedback and suggestions from all staff using transparency (“We do not have all the answers”), avoiding complexity (“We want to unmask simple things that will help”), and asking for any ideas (“There is no such thing as a bad suggestion”). The project team evaluated and discussed all suggestions and then decided which would be tested. Whether a suggestion was abandoned, tested, or adopted, a project team member followed up with each person with a suggestion to explain the group’s decision. Additionally, the project group shared information, data, and results at every huddle and meeting.

We balanced our capacity to meet customer demand by utilizing Heijunka, a method used for scheduling work in a repetitive environment that seeks to level scheduling and pacing, increase visibility, and identify problems early.^4^ We organized workspaces and streamlined inventory using the 5S techniques (sort, simplify, scan, stabilize, sustain),^4^ such as color-coding. We intentionally removed existing hierarchies by having leaders engage staff directly to solve problems and publicizing all improvements, including efficiency and cost reduction data and enhanced ease of work and pain point resolution.

### Operational Definitions and Data Analysis

TAT was the number of calendar days from the device and repair request receipt in CE to the device’s return to service. We analyzed the data using statistical process control (SPC) methods. We defined special cause variation as ≥8 consecutive points above or below the centerline median or mean. To be accepted, such a finding had to be present with a plausible explanation for a system change occurring at that time.^5^ At the project’s start, our existing system could not reliably identify a device’s submission date, which precluded calculating an 8-month baseline TAT. Instead, we identified all devices present in CE at the project start with a submission date and calculated an average TAT for January and February 2017.

We utilized two different approaches to track our progress and process stability. First, because our TAT data was not normally distributed, we tracked the median monthly pump TAT with a run chart for short-term monitoring to identify fluctuations requiring attention and possible modification. For longer-term monitoring, we used a P-chart of the percent of pump repairs completed within 15 days to help us understand pump use and availability to dictate whether we needed to add more pumps and/or more staff to the system. To further evaluate the stability of our system, we generated an x-bar/S chart with a sample of 100 points per month chosen at random. To account for our lack of normal distribution, we performed a logarithmic 10 transformation followed by a retransformation to the original time scale.^5^

### Human Subjects Protection

This quality improvement initiative was considered non-human subjects research and exempt from formal review by the Cincinnati Children’s Hospital Medical Center Institutional Review Board.

## RESULTS

### Process Redesign

Our observations revealed pre-intervention TAT to be long and likely variable due to technicians choosing which devices to assess, which led to inconsistent intake, flow, material ordering, stocking, and output. We decided to adopt a first-in, first-out approach for our entire process to move all devices through the assessment and repair process more efficiently and ensure equal use and wear on all devices. We conducted an intake Kaizen where we found placing all devices on a single rack made it difficult to ensure prompt review of all devices. As a result, we changed our intake racks from any device on any shelf to designated shelves by equipment type. We instituted a daily review of IV pumps to ensure all were triaged and dispositioned. We conducted a triage Kaizen and found labeling inconsistent and difficult to understand. As a result, we revised our repair request forms to ensure customer concerns were clear and information complete and utilized barcode scanning to identify potential failure modes for each device. Rather than starting repairs immediately, we implemented a device repair queue, which reduced our triage process from 30 to 60 minutes to 30 to 60 seconds.

Additionally, organizing devices by racks reduced the space needed for intake and freed up a workstation we used for repair work or triage if demand was high. We conducted work area and throughput Kaizens to assess equipment present at stations and the ways equipment moved through the department. Utilizing the 5Ss,^4^ we stocked stations with the only necessary equipment that was tracked to know when to replenish, and we coded carts used to move equipment through the department either red to denote devices needed repair or green when a repair was complete. A summary of improvement opportunities and interventions is displayed in Table 1. We decreased our productive time slightly (0.6–0.2 days) and decreased lead time by over a month (38.5 days) (Fig. 1B).

### Turnaround Time

**Fig. 2.**
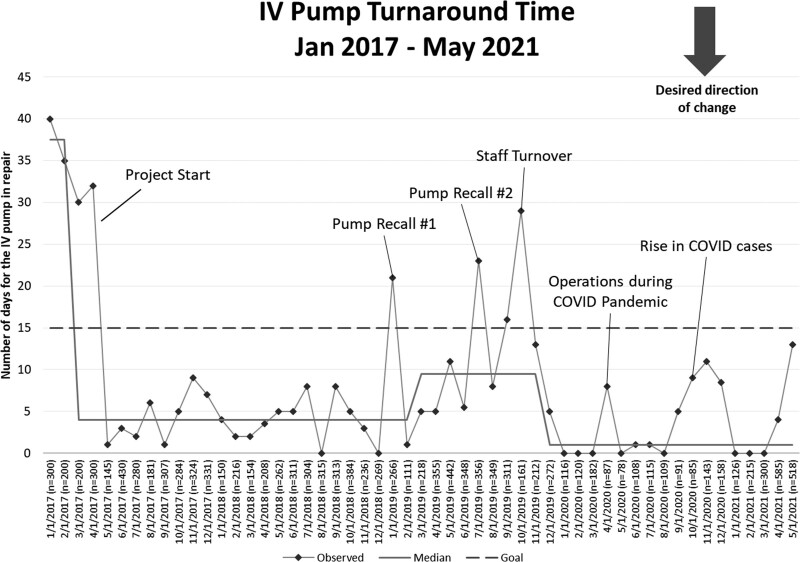
IV pump turnaround time.

Our baseline median TAT for January and February 2017 was 37.5 days. We were unsure what an ideal TAT would be when we began and did not set a specific goal. We found we could turn around pumps during the project within 2 days. However, we identified several devices as part of safety events, requiring a more extensive evaluation before returning into circulation. Returned pumps needed to be recalled, and we had concerns about allowing these pumps into circulation without proper evaluation. In addition, most safety reports are submitted within seven days of an event and often require prolonged evaluation and repair. We, therefore, established a quarantine of any device involved in a safety event for 15 days. As a result, we set our goal at TAT within 15 days of submission to allow for the portion of pumps involved in safety events. Our initial efforts decreased median TAT substantially to 21 days and then to younger than 5 days during May–July 2017. We sustained our median TAT of 4 days for 21 months through February 2019. Median TAT increased to 9.5 days during 2019, which we attributed to increased demand due to two product recalls. During 2020, our TAT decreased to a median of 1 day that we sustained through May 2021 (Fig. 2).

**Fig. 3.**
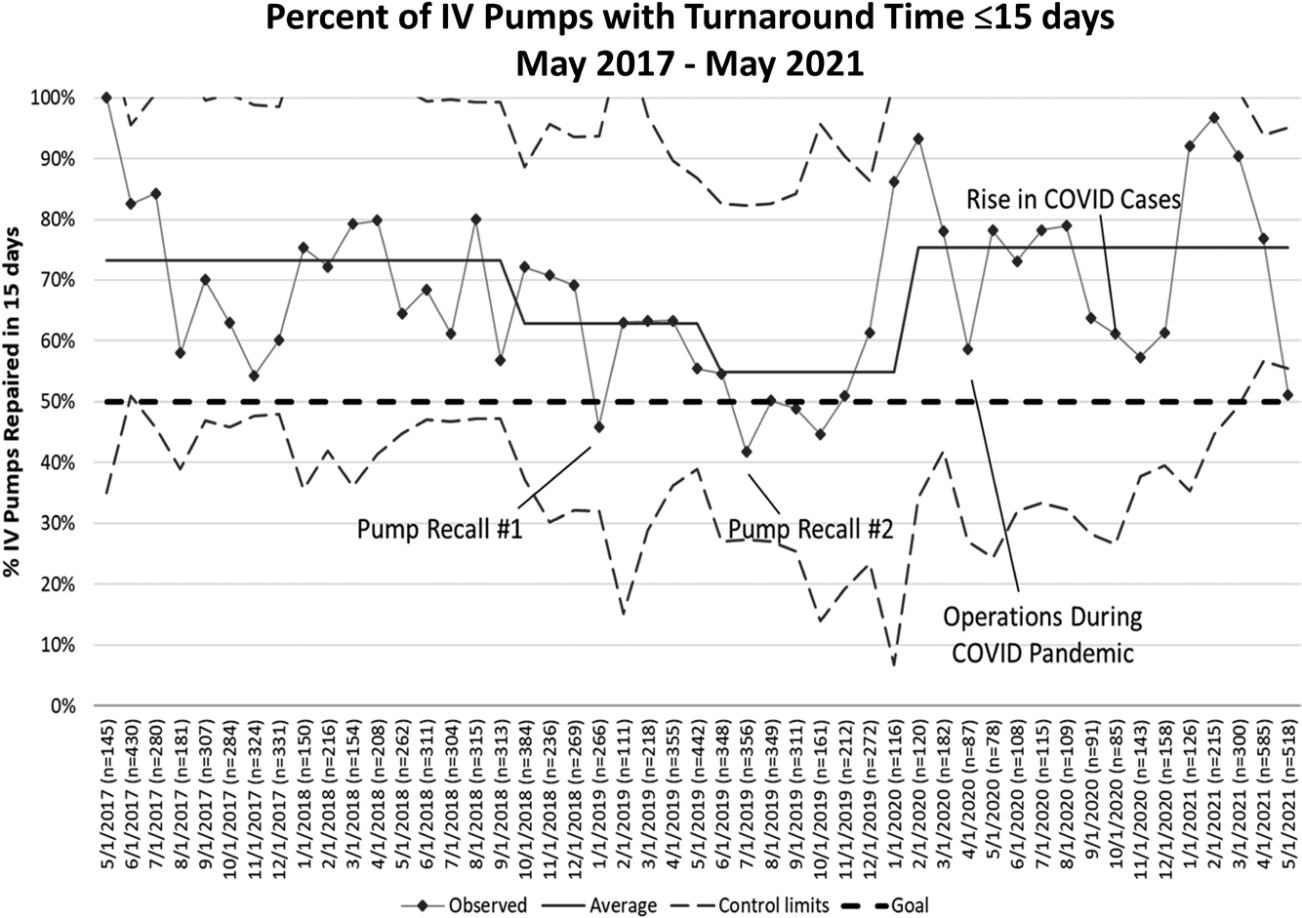
Percent turnaround time less than 15 days.

With our large fleet of pumps and wide distribution of TAT, we utilized the average percent of pumps that met our TAT goal of 15 days and younger. Although the median individual pump TAT measure could detect acute disruptions to our system, this measure would show us when a larger proportion of pumps were present in CE over time, such as with a delay in repair or insufficient staff to maintain throughput. We began tracking this measure at the end of our project once our system was stable and we were looking to sustain our success. We set a goal for 50% of devices to be repaired within 15 days (TAT 15 days and younger), which was chosen based on industry standards. We successfully achieved and sustained our goal for 48 months, with unfavorable centerline shifts in October 2018 and June 2019 attributed to product recalls, staff turnover, and a return to baseline in February 2020 (Fig. 3). Our x-bar/S chart analysis system demonstrated a stable system until early 2019, corresponding with the two recalls, our increased staff turnover, and the COVID-19 pandemic. The system returned to baseline with rolled-back pandemic precautions in late summer 2020. (**See Figures, Supplemental Digital Content 1,** http://links.lww.com/PQ9/A395.)

Product recalls increased TAT by requiring all recalled pumps to be evaluated and repaired, which increased workload and decreased efficiency for other repairs. In addition, the COVID-19 pandemic affected our system in many ways, likely common to many healthcare and non-healthcare contexts. Specifically, we experienced staff loss and turnover, supply chain delays, and a need for constant adjustments to staffing and function based on reassignments to pandemic response, requirements to maintain low staff numbers on-site and a lack of co-op students. Throughout, our median TAT was one day, with bumps that did not rise to special cause in the late 2020 and mid-2021.

## DISCUSSION

We successfully revamped and improved our IV pump maintenance process using Lean principles. As a result, we reduced TAT by 89% following our Lean project, sustained at least a 74% reduction for four years, and recently achieved a 97% reduction from our original baseline. Similarly, we sustained our percent pumps with TAT 15 days and younger at greater than 50% for 4 years. Large-scale recalls, and staff turnover have challenged our system, but we maintain an efficient process.

The COVID-19 pandemic impacted our department, like in many other areas, but our TAT remained stable and improved compared to our prepandemic baseline. This stability could be due to lower volumes and, thus, greater efficiency. For example, average monthly repairs during 2017–2019 ranged from 260 to 283, whereas in 2020, they were 116. Another possibility is that we struggled to maintain a fully trained staff when many staff retired or resigned and newly hired staff did not stay long.^6^ This could have led to inaccurate tracking. For example, instead of opening a ticket when repairs began, a ticket was both opened and closed when repairs were complete, giving an artificial appearance of rapid TAT. We know of this occurring during 2020, but we cannot be certain if it happened often enough to drive down our median TAT. Anecdotally, we can also state that we did not experience overcrowding or increased complaints during the same period, suggesting our system continued to function well.

Our short- and long-term success was largely due to our team’s commitment to Lean principles and methods. Historically, Lean’s impact in healthcare has been in short-term gains,^7–10^ whereas long-term improvement and sustainability have been less clear.^11,12^ This observation may be because, in most healthcare systems, an individual “siloed” focus is the standard and conflicts with Lean’s goal to involve all operators in intervention design and execution, independent of hierarchy.^13^ We intentionally addressed possible hierarchal barriers through transparency, frequent presence in the department and during discussions, and universal feedback for all suggestions, regardless of their result. It is also likely that our effort to build group dynamics and collaboration in our initial project enabled us to sustain improvements despite the challenges of large-scale device recalls and an unusually high rate of staff turnover in the late 2019.

We translated success in corrective maintenance to success in preventive maintenance. Briefly, we changed our approach from specific devices receiving quarterly maintenance, regardless of the history of corrective maintenance, to accounting for time since the last preventive maintenance whenever a pump was in CE. This change allowed us to distribute the effort better and maintain our fleet, which likely helped keep our corrective maintenance process stable. When we finished our corrective maintenance project, we shifted to our next priority rather than building and executing a control plan. We did not develop a specific control plan because we did not need one. Similarly, rather than defining thresholds for action or modifying our process, we monitored for acute change. We mitigated them in real time, using our P-chart as an indicator of overall stability. As a result, we were fortunate to respond to the recalls by bringing manufacturer staff on-site to complete the maintenance. We also responded to staff turnover while achieving our goal of 50% of pumps returned within 15 days.

### Limitations

Our study is subject to several limitations. First, our study took place at a single institution, which may limit its generalizability. As with any improvement project, context is important; institutions and groups may not have the same precedent, structure, or support.^14^ Maximizing quality and minimizing cost for medical devices can be challenging, and multiple approaches have been proposed and/or tested.^15^ Our sustained improvements may indicate that a similar approach could work in many facilities’ CE departments. Second, we could not calculate baseline TAT based on historical performance. Although not required, a baseline is typically determined using a period before the project starts. Using a convenience sample at the project start may not reflect the true baseline. However, although we cannot say to what degree, we can confidently say that we likely improved over baseline. Third, our systems lacked the granular detail necessary to conduct an adequate cost analysis. We estimate our initial project was cost-saving by canceling further purchasing and staff growth that had been planned, but we cannot be certain. As a result, we cannot determine whether sustaining our system was cost-effective. Finally, our experience data are anecdotal. We could not retrospectively assess the volume or nature of complaints or repair requests because we did not have an adequate tracking system. We can say that we had few complaints following the project, although we cannot say with certainty it was less than baseline.

## CONCLUSIONS

We utilized Lean methodology to transform our IV pump repair process, which we sustained for 4 years without significant modification or intervention. Our successfully sustained improvement suggests that long-term sustainability of Lean-driven improvements in healthcare is possible.

## DISCLOSURE

The authors have no financial interest to declare in relation to the content of this article.

## ACKNOWLEDGMENTS

Assistance with the study: Members of the Clinical Engineering Department and Lean Collaborative at Cincinnati Children’s Hospital Medical Center. Statistical analysis help from Amy M Anneken and Dr. Denise L White.

## Supplementary Material


