# Supplementary figures and images for: Sustained Reduction in Intravenous Pump Turnaround Time Using Lean Methodology
Source: Pediatr Qual Saf. 2022 Aug 1;7(4):e585. doi: 10.1097/pq9.0000000000000585 (PMC9345645; doi:10.1097/pq9.0000000000000585)

### IntraVenous (IV) Pump Turnaround Time (May 2017 – May 2021)

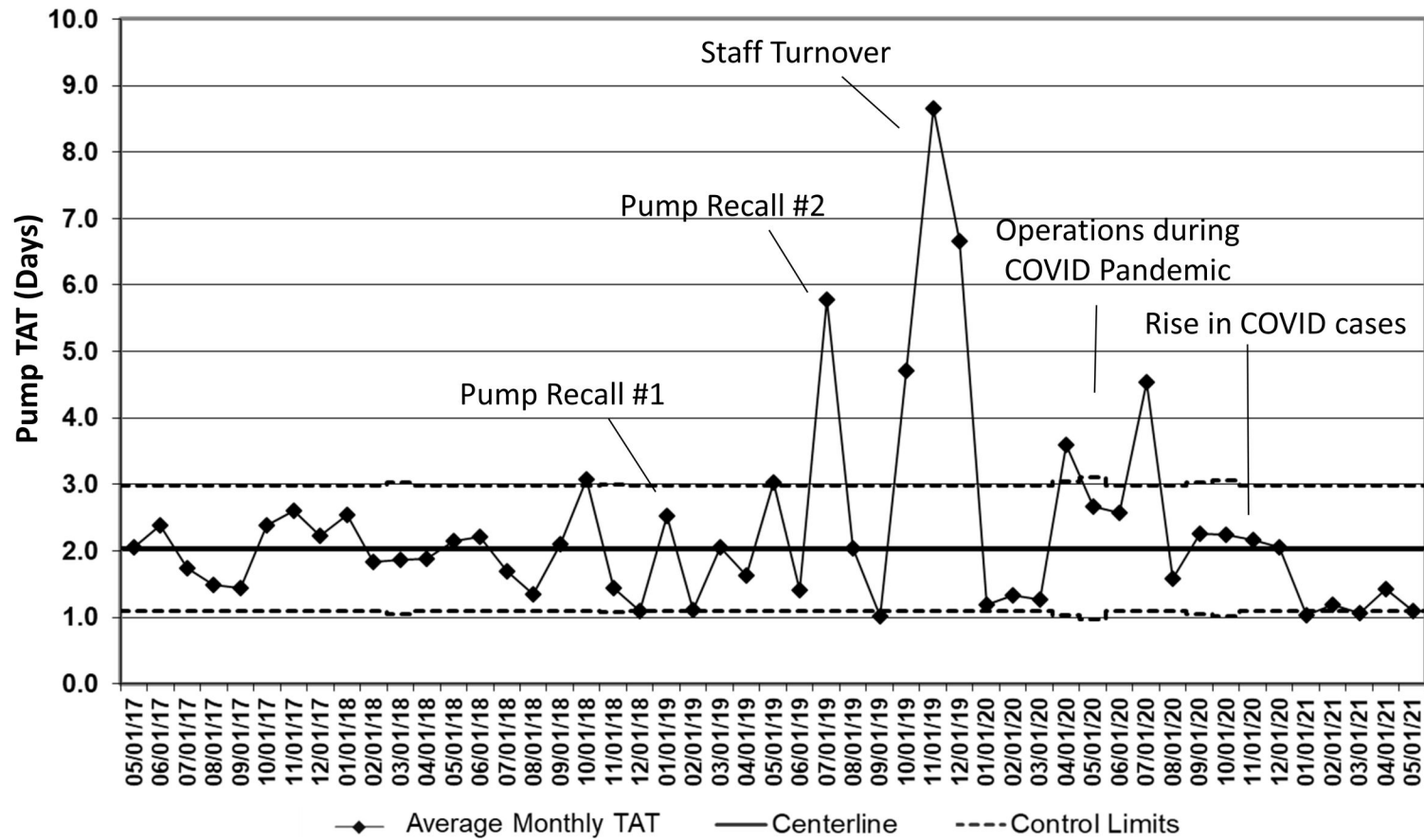

### IntraVenous (IV) Pump Turnaround Time (May 2017 – May 2021)

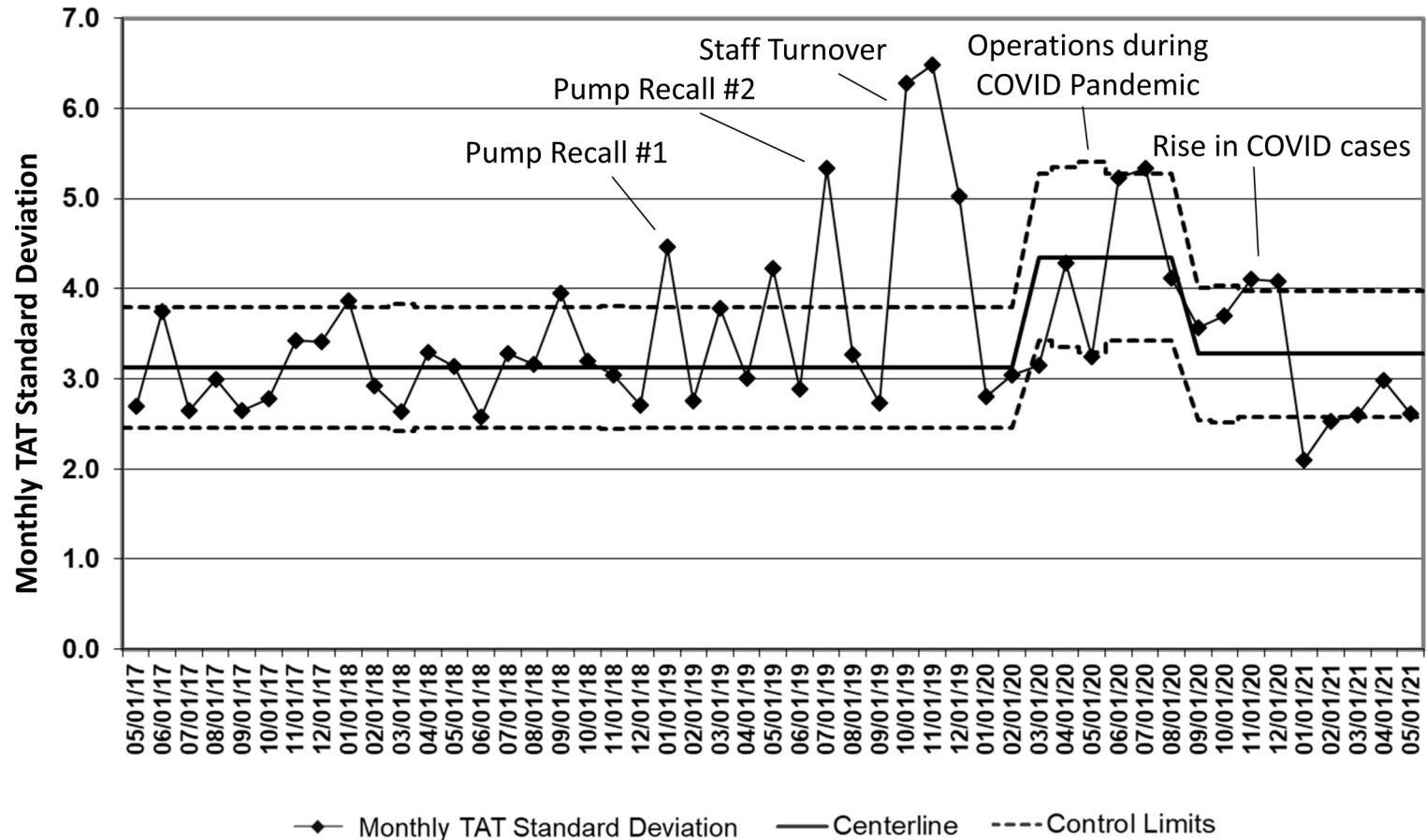

Supplement: Supplementary file 1 [file pqs-7-e585-s001.pdf]
